# Sample Stacking–Capillary Electrophoretic Analysis of Nitrate and Nitrite in Organic- and Conventional-Originated Baby Food Formulas from Turkey

**DOI:** 10.1021/acsomega.2c07969

**Published:** 2023-01-25

**Authors:** Nigar Kamilova, Zeynep Kalaycıoğlu, Ayşegül Gölcü

**Affiliations:** Faculty of Science and Letters, Department of Chemistry, Istanbul Technical University, Istanbul34469, Turkey

## Abstract

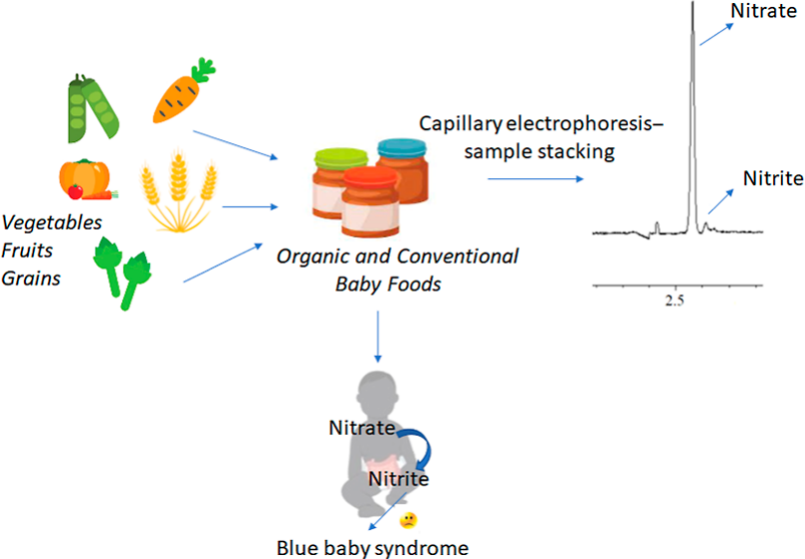

Commercially available baby food formulas represent a convenient alternative to homemade meals especially in the recent years. The main purpose of this study is the determination of nitrate and nitrite levels by a sample stacking–capillary electrophoresis technique in the baby foods. The baby foods analyzed were organic-originated, vegetable-based, fruit-based, mixed puree, and a vegetable soup. Vegetables and fruits have high nitrate and nitrite concentrations. Nitrate itself is not actually hazardous. However, nitrite has negative health effects. Thus, baby foods have to be strictly controlled due to the potential health risk of nitrite. In this study, the sample stacking method enhanced the sensitivities of both anions. Nitrate contents ranged from 16.1 to 285 mg/kg with a mean concentration of 149 mg/kg for all samples. The lowest nitrate amount belonged to red fruity milky baby food whereas the highest nitrate was found in organic pumpkin, banana, and carrot mixed puree. The nitrite levels in all the samples were below the LOQ value of the analyzed method. As a conclusion, there is no health risk of the analyzed baby foods regarding nitrate and nitrite levels considering the regulations.

## 1 Introduction

Vegetables, fruits, and processed meats are the main sources of dietary nitrate and nitrite.^1^ In cured and processed meats, nitrate and nitrite ions are added as food additives to prevent the growth of bacteria. Vegetables have high nitrate concentrations whereas nitrite levels are comparatively lower.^2^ Actually, natural fruits and vegetables account for a huge majority of nitrate and nitrite consumption rather than food additives.

Nitrate is not to be considered as hazardous. However, commensal bacteria in the mouth and gastrointestinal system convert nitrate into nitrite. Nitrite can be then turned into nitrosamines, which are thought to be carcinogenic. Additionally, it is asserted that children under the age of 6 months who consume nitrite develop methemoglobinemia called as blue baby syndrome. On the other hand, the advantages of dietary nitrite and nitrate are currently receiving more attention from science and medicine.^3,4^ Breast milk contains higher levels of nitrate and nitrite than the majority of commercial products. According to a report, breast milk’s nitrite and nitrate levels aid in the growth and development of the newborn.^5^ The current literature indicates the beneficial benefits of dietary NO_3_^–^ supplementation on the physiological performances of older persons.^6^

Considering the potential risks of nitrate and nitrite to baby and infant health, it is advised against giving newborns under 3 months of age spinach, beets, green beans, carrots, squash, and other vegetables with high nitrate contents.^7^ Because baby foods are a significant source of nutrients and a special supply of food during the first few months of life, they serve specific purposes in newborns’ diet. It is clear that the newborn and infant food industry has to show special care. Moreover, regulatory agencies of different counties establish limits for nitrate and nitrite in newborn and infant formulations. Thus, it is important to evaluate the quality control of baby foods in a fine-tuned way.

Numerous research have been conducted with the goal of quantifying nitrate and nitrite in different baby foods from worldwide by HPLC,^8^ spectrophotometry,^9,10^ flow injection analysis,^11^ and capillary electrophoresis.^12^ In these studies, organic and conventional baby foods; animal-based, plant-based, and mixed-origin infant foods; and vegetable, cereal, fruit, and milk-based commercial baby foods were evaluated.

Our study aims to determine nitrate and nitrite levels in vegetable, fruit, and grain-based baby foods. A capillary electrophoresis (CE) technique, which is fast and economic, was used in this study. In the CE technique, the analyte is injected into the capillary column in only a few nL, which is the most notable characteristic of CE. The detection limits were improved by sample stacking technique without any sample preconcentration. Sample stacking technique is one of the most effective ways of increasing peak sensitivity in capillary zone electrophoresis.^13^ In this online sample preconcentration technique, the injection volume of sample is increased. Because high-volume injection results in overloaded peaks, the conductivity of the separation buffer is increased. A lower electrical field than that of the sample zone is presented by higher conductivity in the separation buffer. When both the anions reach the buffer region with high conductivity while still moving quickly in the sample zone, the electric field falls and the velocity of the anions slow down. As a result, the sample condenses along the boundary between the sample and buffer zones and is seen as sharp peaks in the detector.

This method can be proposed as a powerful technique for the routine analysis of nitrate and nitrite anions in different originated baby and infant food samples.

## 2 Results and Discussion

### 2.1 CE Analysis of Nitrate and Nitrite

In order to analyze the nitrate and nitrite, the capillary electrophoretic method which was developed by Kalaycıoğlu and Erim (2016) was performed.^14^ Successful results for different matrices such as fish products,^14^ different honey varieties,^15^ and bee pollen^16^ were obtained with this method by our group before.

The applied CE method is based on the reduction in electroosmotic flow (EOF) to the cathodic side caused by the migration of nitrite and nitrate anions through a separation medium at low pH values. When injected from the cathodic side, nitrate and nitrite anions rapidly move across reduced EOF due to their strong electrophoretic mobilities. The mobility of nitrate anion is not affected by the pH of the medium. However, pH has an impact on the electrophoretic mobility of nitrite which is a conjugate base of a weak acid. Because the pKa value of the nitrous acid is 3.15, the pH of the separation medium was chosen quite closely to this value. The movement of both ions against the EOF would be challenging because increasing the pH of the separation medium will hasten the EOF in the capillary. For this reason, formic acid/sodium formate was selected as the background electrolyte. Based on both the arrival times of the anions and resolution between nitrate and nitrite, the optimum pH value was chosen as 4.0. At this pH, both ions were negatively charged and moved quickly through the capillary against the EOF.

In the preliminary experiments, a small-volume sample injection technique was applied to baby food samples. However, the amount of both nitrate and nitrite in the samples were found to be under the limit of detection (LOD). In this study, 30 mmol/L sodium sulfate was added into the formic acid–sodium formate buffer in order to enhance its conductivity. The detection limits of both ions are dramatically lowered by this sample stacking technique.

**Figure 1:**
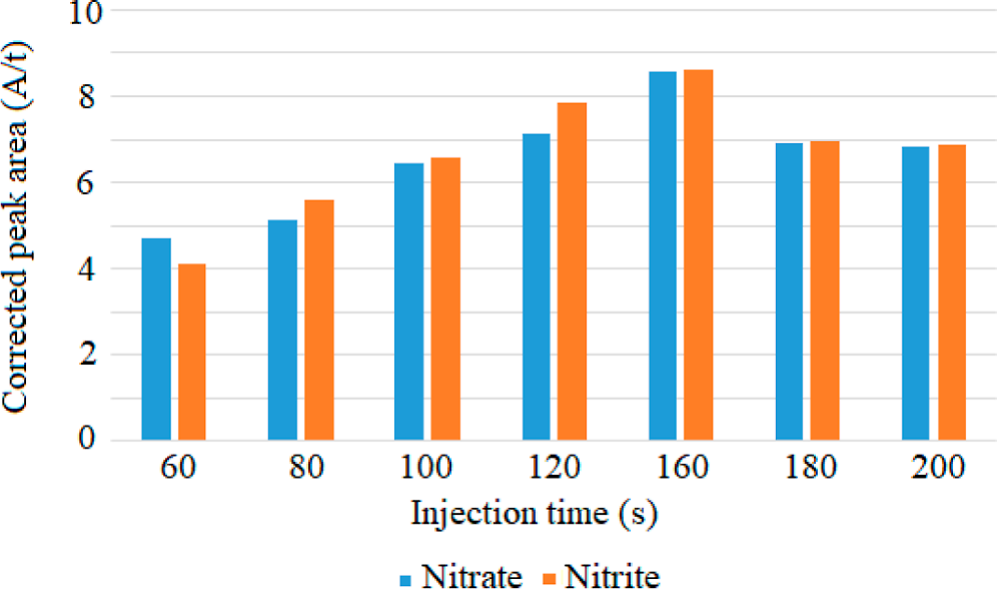
Optimization of injection times for nitrate and nitrite.

The optimized separation buffer consisted of 30 mmol/L formic acid–sodium formate buffer containing 30 mmol/L sodium sulfate. The pH of the buffer solution was adjusted to 4.0 with 0.1 mol/L NaOH solution. In order to determine the optimum injection time, nitrate and nitrite anions were injected at 50 mbar pressure, at different times between 60 and 200 s. Undesired peak broadening occurred for both anions when the injection time exceeded 200 s. As seen from Figure 1, the highest corrected peak area for both nitrate and nitrite anions were achieved with an injection time of 160 s. Therefore, the optimum injection time was determined as 160 s.

**Figure 2:**
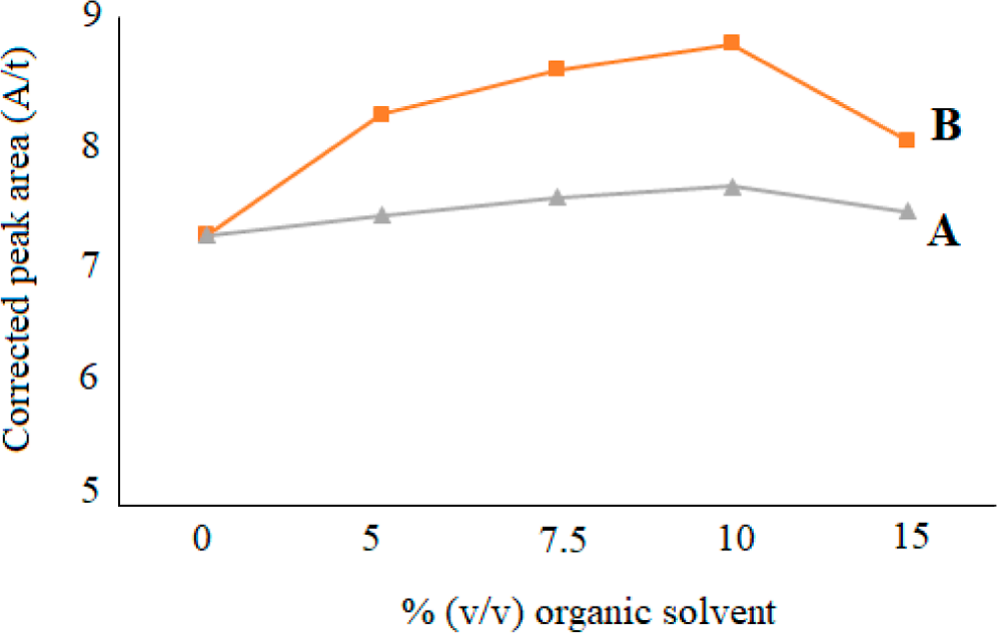
Optimization of organic solvent contents for nitrate anions in a representative baby food sample (A: methanol and B: acetonitrile).

In the sample stacking method, the conductivity of the sample region is reduced and a high electric field is provided in this region. When the electric field is increased in the sample field, the peak area remains higher. Thus organic solvents bring along a stacking effect compared to that for aqueous buffers. In this study, the effect of organic solvents such as acetonitrile and methanol, which are known to have low conductivity was investigated on the detection limits. Methanol and acetonitrile were separately added into the sample extracts in volumes ranging from 5 to 15% (v/v). Methanol had no effect on peak height and peak shapes as seen from Figure 2A. On the other hand, in the presence of 10% acetonitrile, the peak areas of both anions were increased and so the detection limits were decreased. Addition of more than 10% acetonitrile caused decrease in the peak areas (Figure 2B). Because nitrite was not detected in all baby food samples, the effect of methanol and acetonitrile was only monitored for nitrate anions.

**Figure 3:**
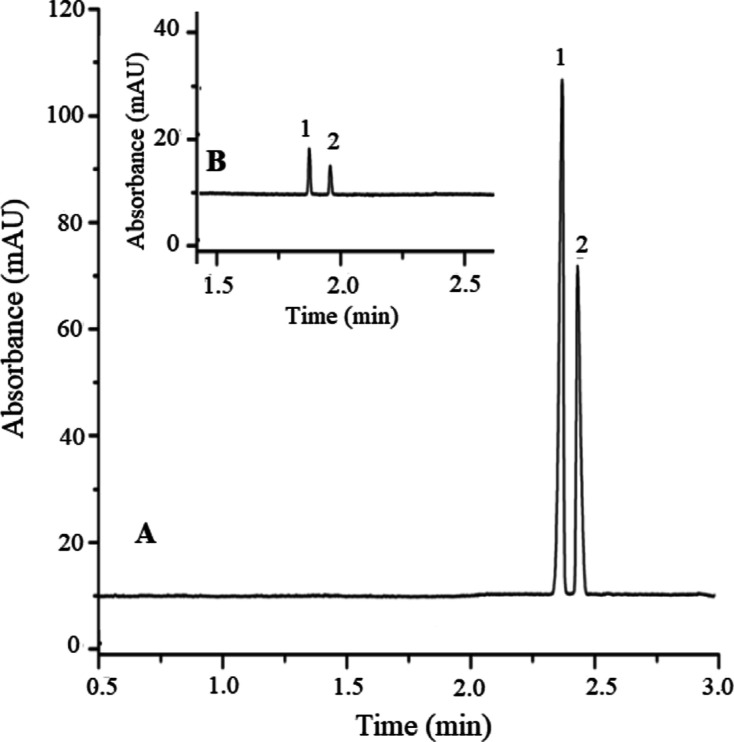
Comparison of the sample stacking experiment and small-volume injection of a standard sample containing 100 μmol/L NO_3_^–^ and NO_2_^–^. Injection: (A) 50 mbar, 160 s and (B) 50 mbar, 6 s. Running potential, −25 kV; 210 nm. Peaks, 1: NO_3_^–^, 2: NO_2_^–^.

Nitrate and nitrite anions were injected in both small-volume injection mode and sample stacking mode optimized. The comparison of these two modes can be seen in Figure 3. It was seen that the stacking mode increased the detection sensitivity of both anions by 30 times.

The analytical performance of the method is given in Table 1. Nitrate and nitrite calibration curves were constructed in the range of 1.5–50 and 1.5–30 μmol/L, respectively. The correlation coefficient was 0.998 for both the anions. Acetonitrile was added to the solutions at a final concentration of 10% (v/v) and vortexed for 10 s. Peak areas were calculated by injecting the each anion for 160 s with the CE–sample stacking technique. The corrected peak area was calculated by dividing the peak areas by the migration time of the peaks. Calibration curves were drawn from the obtained corrected peak areas.

**Table 1:** Analytical Performance of the Proposed Method

**Parameter**	**Nitrate**	**Nitrite**
**Intra-day precision**(*n* = 5)		
corrected peak area (RSD, %)	4.21	4.93
migration time (RSD, %)	2.13	3.85
**Inter-day precision**(*n* = 15)		
corrected peak area (RSD, %)	4.62	4.71
migration time (RSD, %)	3.16	4.12
**Linearity**		
linear range (μmol/L)	1.5–50	1.5–30
regression equation	*y* = 0.0076*x* – 0.0016	*y* = 0.0043*x* + 0.0014
correlation coefficient	0.998	0.998
**LOD** (**μg/mL**, based on the analytes)	0.028	0.021
**LOQ** (**μg/mL**, based on the analytes)	0.093	0.070
**LOD** (**μg/mL**, based on the sample)	0.121	0.097
**LOQ** (**μg/mL**, based on the sample)	0.403	0.323

The precision of the method was evaluated by measuring its intra-day and inter-day reproducibilities. Intra-day reproducibility was determined by sequential injections of nitrate and nitrite anions five times on the same day. Inter-day reproducibility was determined by five injections of both anions for 3 different days (3 days × 5 injections). Percent relative standard deviation (RSD %) values of the corrected peak areas (*A*/*t*) and migration times (*t*) of the anions were calculated. As seen from Table 1, RSD % values for both anions are lower than 5.93. According to the values found, the precision of the method was found to be appropriate.

The LOD value was calculated as three times the average noise taken from two different baseline areas, and the LOQ (limit of detection) value was calculated as 10 times. The LOD values for nitrate and nitrite were 0.027 and 0.021 μg/mL, respectively. LOQ value for nitrate was found to be as 0.093 μg/mL whereas LOQ for nitrite is 0.070 μg/mL.

Standard nitrate and nitrite analytes were added into a baby food sample to determine the recovery of the method. Three different concentrations of standard nitrate solution were added at concentrations corresponding to 50, 100, and 200% of the real sample concentration. The percentage of recovery was calculated with the following formula
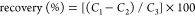
where *C*_1_ is the concentration determined in the fortified sample, *C*_2_ is the concentration determined in the unfortified sample, and *C*_3_ is the concentration of the added standard. The recovery results of the method were found between 88 and 104% as seen in Table 2.

**Table 2:** Recovery Values of Nitrate for a Baby Food Sample at Three Concentration Levels

**Analyte**	**Sample content (μg/mL)**	**Anion added (μg/mL, *n* = 3)**	**Anion found (μg/mL, *n* = 3)**	**Recovery (%) ± SD(μg/mL, *n* = 3)**
nitrate	11.10	5.55	14.65	88.0 ± 4.0
		11.10	20.33	91.6 ± 2.8
		22.20	34.63	104 ± 9
nitrite		5.55	5.24	94.4 ± 3.1
		11.10	10.82	97.5 ± 4.8
		22.20	21.74	97.9 ± 4.2

### 2.2 Determination of Nitrite and Nitrate Concentrations in Baby Food Samples

The conditions for the extraction of nitrate and nitrite from baby food samples were optimized. As seen from Figure S1A (see Supporting file), the sensitivity for nitrate was increased after 15 min of magnetic stirring as compared to 30 min duration. The highest nitrate peak was obtained after 30 min in ultrasonic bath (Figure S1B). Thus, the suspensions were stirred at 60 °C for 15 min on a magnetic stirrer. Finally, it was kept in an ultrasonic bath for 30 min.

**Figure 4:**
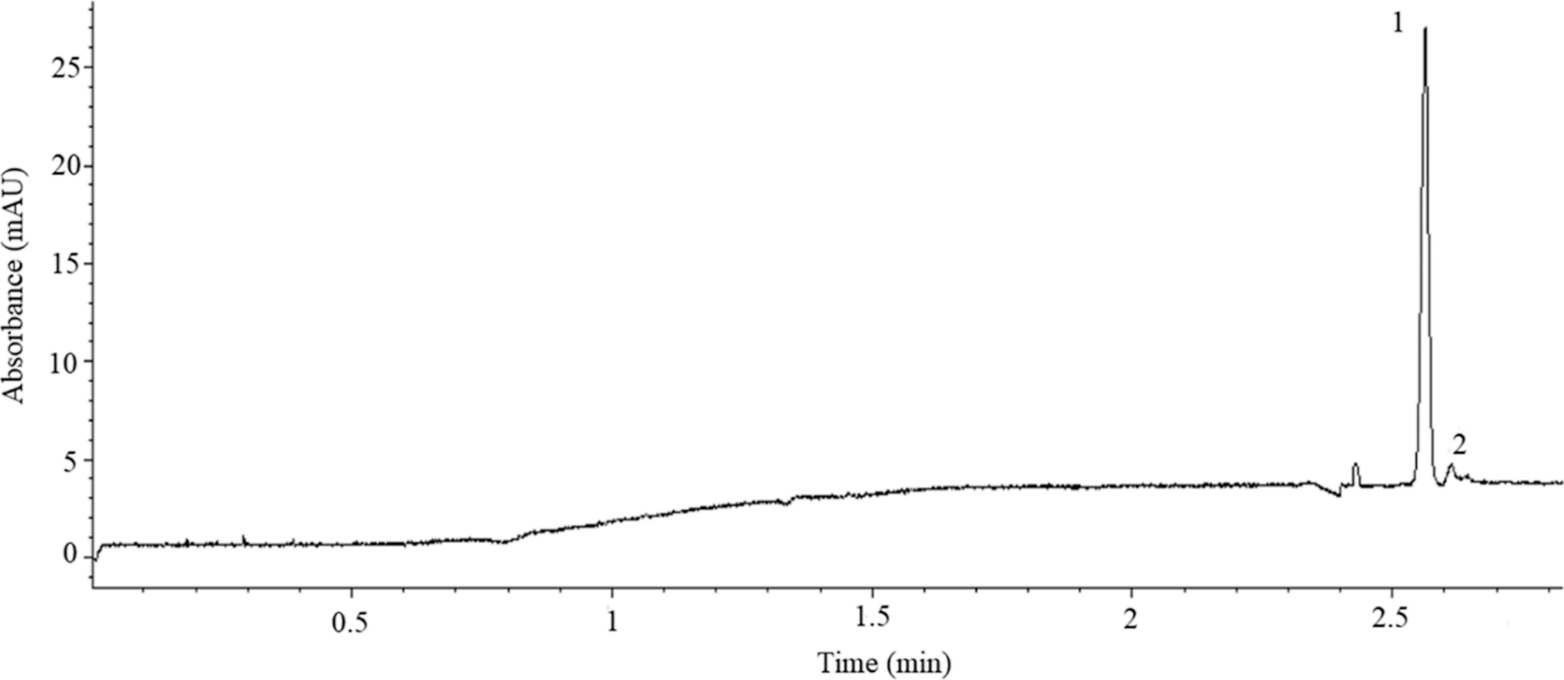
Electropherogram of a baby food sample. Injection: 50 mbar, 160 s. Running potential, −25 kV; 210 nm. Buffer: 30 mmol/L formic acid, 30 mmol/L Na_2_SO_4_, and pH = 4.0. Peaks, 1: NO_3_^–^, 2: NO_2_^–^.

Seven different baby food formula samples were applied to the CE–sample stacking method in order to determine the nitrate and nitrite concentrations. Quantitative analysis of both anions was performed by the external standard calibration method. A representative electropherogram of a baby food sample is given in Figure 4.

Nitrate and nitrite contents in baby foods are given in Table 3. The nitrate anion has been found in all baby food formulas in amounts ranging from 16.1 to 285 mg/kg. The baby food sample with the highest nitrate content includes pumpkin, banana, and carrot. This sample was followed by broccoli soup with bone broth with a nitrate content of 235 mg/kg. The samples with the lowest nitrate content are organic cereal-based supplementary food (13.7 mg/kg) and a baby food with red fruits (16.1 mg/kg). The nitrite anion is below the LOQ in all baby food samples. Nitrate concentrations in baby foods were below the legal limit (200 mg/kg) of foodstuffs, including the baby foods, set by Turkish regulations.^17^ Moreover, nitrate amounts were below the legal upper limits set by the European Union.^18^

**Table 3:** Nitrate and Nitrite Contents in Baby Food Samples

**Brand**	**Ingredient of baby food**	**Nitrate ± SD**	**Nitrite**
brand A	organic grain-based supplement	13.7 ± 1.8	<LOQ
brand B	Eight grain baby food with milk and apple	119 ± 5	<LOQ
brand B	milky fruity eight cereal supplement	167 ± 7	<LOQ
brand C	milky fruity eight cereal supplement	213 ± 4	<LOQ
brand D	mixed cereal breakfast	16.1 ± 1.2	<LOQ
brand E	red fruity milky	285 ± 16	<LOQ
brand F	organic pumpkin, banana, and carrot mixed puree	235 ± 10	<LOQ

The literature contains less information on the nitrate and nitrite levels of baby foods (Table S1) (see supplementary file). Vasco and Alvito (2011) reported the nitrate concentration of organic and conventional baby foods by an HPLC method.^8^ The LOD for nitrate was found to be as 0.1 μg/mL (1 mg/kg). Vegetable-based baby foods had an average nitrate content of 102 mg/kg, whereas fruit juices and purees had a median nitrate content of 5 mg/kg. Only one sample made with vegetables contained more nitrate than the legal maximum (200 mg/kg). The nitrate and nitrite contents of 104 baby food with animal, plant, and mixed origins were investigated using the spectrophotometric technique by Cortesi et al. (2015).^9^ Plant-originated samples showed the highest average nitrate content (45.5 mg/kg), followed by animal-originated samples (27.39 mg/kg), and finally mixed-origin samples (24.19 mg/kg). The mean nitrite concentrations were reported in baby foods as 14.82, 12.48, and 8.2 mg/kg for animal origin, mixed origin, and plant origin, respectively. Chetty and Prasad (2016) reported the levels of nitrite and nitrate in commercial baby foods based on vegetables, cereal, fruit, and milk from Fiji.^11^ Nitrate levels ranged from 8.00 to 220.67 mg/kg while nitrite was only found in a sample made with vegetables. The LOD for nitrate was 0.040 μg/mL. In only one capillary electrophoretic study in the literature,^12^ 14 baby food samples from Brazil were analyzed for their nitrate and nitrite contents. Nitrate levels were found be between 8.44 (banana- and milk-based puree) and 247.70 (organic pumpkin-based puree). Nitrite contents of all samples were below the LOQ of the method. The LOD values were 0.09 and 0.15 mg/L for nitrate and nitrite, respectively. Erkekoğlu and Baydar (2009) evaluated only the nitrite contents in milk-based, cereal-based, vegetable-based, and fruit-based baby foods and infant formulas from Turkey.^10^ In 42 samples, the average nitrite contamination was found to be 204.07 μg/g, with a maximum of 1073 μg/g using the spectrophotometric technique. As seen from Table S1, the lowest LOD values for both anions were found in our report among these studies.

## 3 Materials and Methods

### 3.1 Chemical and Standard Solutions

Formic acid, potassium nitrate, sodium nitrite, and sodium hydroxide were purchased from Merck (Darmstadt, Germany). Acetonitrile was from J. T. Baker (Deventer, Netherlands). All solutions were prepared with deionized water obtained with the Elga PURELAB Option-7-15 model system.

Nitrate and nitrite stock standard solutions were prepared in deionized water at a concentration of 10 mmol/L. The solutions were stored in a refrigerator at 4 °C until analysis. Calibration solutions were also prepared from these stock solutions by diluting them with deionized water.

### 3.2 Baby Food Samples

Seven different baby food samples of six brands (brand A–organic grain-based supplement *n* = 1, brand B–eight grain baby food with milk and apple, *n* = 1 and milky fruity eight cereal supplement *n* = 1, brand C–mixed cereal breakfast *n* = 1, brand D–red fruity milky *n* = 1, brand E–organic pumpkin, banana, and carrot mixed puree *n* = 1, and brand F–broccoli soup for babies *n* = 1) were purchased from a local store in İstanbul, Turkey. The samples were kept at 4 °C until the analysis.

### 3.3 Sample Preparation

All the baby food samples except the organic pumpkin, banana, and carrot mixed puree and broccoli soup were already powdered. The puree and the soup samples were also homogenized. One hundred mg of each sample was carefully weighed and added into the tubes containing 10 mL of deionized water at 60 °C. The tubes were sealed and vortexed for 1 min. The nitrate and nitrite were extracted during 15 min on a magnetic stirrer and after ultrasonic bath for 30 min. After cooling, the suspensions were passed through Whatman No: 41 filter paper and the volume of the extracts were made up to 10 mL with deionized water. An aliquot of sample extract was filtered with a 0.45 mm pore size regenerated cellulose membrane filter. One hundred μL of ACN was added into an injection vial containing 900 μL of sample extract. The vial was vortexed for 10 s and directly injected into the CE system.

### 3.4 Apparatus and Operating Conditions

An Agilent 1600 capillary electrophoresis system (Waldbronn, Germany) was used for the analysis. The data processing was carried out with Agilent ChemStation software. Separations were performed in silica capillaries with 50 mm i.d. (Polymicro Technology, Phoenix, AZ, USA). The total length of the capillary was 58 cm, and the length to the detector was 50 cm. The new fused silica capillary was conditioned prior to use by rinsing with 1 mol/L NaOH for 30 min and with water for 10 min. The capillary was flushed with 0.1 mol/L NaOH for 2 min, water for 2 min, and buffer for 5 min between runs. The temperature was set at 25 °C. Sample injections were made at 50 mbar for 160 s. The applied voltage was −25 kV and the UV detection was carried out at 210 nm.

## 4 Conclusions

As the first year of life is crucial for a child’s development, the composition and quality of commercial baby food must be controlled to reduce the risk of exposure to nitrate and nitrite. In this study, nitrite and nitrate contents of seven baby food samples from the Turkish market were determined using a simple, rapid, sensitive, and efficient CE–sample stacking technique. Vegetables containing baby food and broccoli soup have significantly higher nitrate contents among all baby food samples investigated. Nitrite levels were found to be under the LOD of the method.

## Supporting Information Available

Conditions for the extraction of nitrate and nitrite from baby food samples and the literature information on the nitrate and nitrite levels of baby foods (PDF)
