# Supplementary material for: Sample Stacking–Capillary Electrophoretic Analysis of Nitrate and Nitrite in Organic- and Conventional-Originated Baby Food Formulas from Turkey
Source: ACS Omega. 2023 Jan 25;8(5):5097–102. doi: 10.1021/acsomega.2c07969 (PMC9909805; doi:10.1021/acsomega.2c07969)
Supplement: Supplementary file 1 — ao2c07969_si_001.pdf [file ao2c07969_si_001.pdf]

Sample Stacking-Capillary Electrophoretic Analysis of Nitrate and Nitrite in Organic and Conventional Originated-Baby Food Formulas from Turkey

Nigar Kamilova[1], Zeynep Kalaycıoğlu[1*], Ayşegül Gölcü[1*]

[1] Istanbul Technical University, Faculty of Science and Letters, Department of Chemistry, Istanbul, Turkey

*E-mail: aysgolcu@itu.edu.tr, https://orcid.org/0000-0001-5228-1682

*E-mail: kalaycioglu z@itu.edu.tr, https://orcid.org/0000-0002-0967-0997

Supporting Information

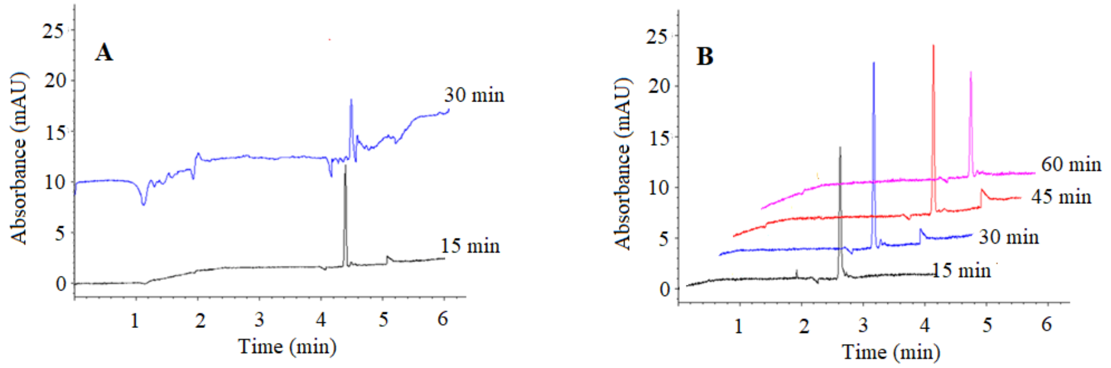

Figure s1: Optimization of **A:** magnetic stirring time and **B:** ultrasonic bath time

Table s1: Results for the determination of nitrite and nitrate in baby food samples.

Sample	Nitrate (mg/kg)	Nitrite (mg/kg)	Method/LOD, µg/mL	Ref.
80 baby foods (commercial baby food labeled as from organic or conventional origin)	7 – 108	-	HPLC/0.1 µg/mL	(8)
104 infant foods (animal origin, plant origin, mixed origin)	0.35 – 131.68	1.12 – 80.22	Spectrophotometric/NG	(9)
42 baby food sample (milk-based, cereal-based, vegetable-based, and fruit-based)	-	nd – 1073	Spectrophotometric/25 µg/mL for nitrite	(10)
108 baby food (vegetable-based, fruit-based, milk-based, cereal-based, rice-based)	8.00 – 220.67	<LOD − 0.55	FIA/0.040 µg/mL	(11)
14 baby food samples (vegetable-based, meat-based,chicken-based, pasta-based, fruit-based purees)	8.44 – 247.70	<LOD	CE/0.09 µg/mL for nitrate and 0.15 µg/mL for nitrite	(12)
7 baby foods (vegetable-based, fruit-based, mixed-puree and a vegetable soup	13.7 – 285	<LOQ	CE-sample stacking/0.028 µg/mL for nitrate and 0.021 µg/mL for nitrite	This study
